# Electromagnetic Absorption and Mechanical Properties of Natural Rubber Composites Based on Conductive Carbon Black and Fe_3_O_4_

**DOI:** 10.3390/ma15196532

**Published:** 2022-09-21

**Authors:** Pornlada Pongmuksuwan, Kiadtisak Salayong, Titipong Lertwiriyaprapa, Wanlop Kitisatorn

**Affiliations:** 1 Department of Materials and Production Technology Engineering, Faculty of Engineering, King Mongkut’s University of Technology North Bangkok, 1518, Pracharat 1 Road, Wongsawang, Bangsue, Bangkok 10800, Thailand; 2 Research Center of Innovation Digital and Electromagnetic Technology (iDEMT), Department of Teacher Training in Electrical Engineering, Faculty of Technical Education, King Mongkut’s University of Technology North Bangkok, 1518, Pracharat 1 Road, Wongsawang, Bangsue, Bangkok 10800, Thailand

**Keywords:** natural rubber foam, electromagnetic wave absorber, conductive carbon black, Fe_3_O_4_

## Abstract

In contemporary civilization, the electromagnetic radiation from electronic devices and communication systems has become a substantial pollutant. High-performance electromagnetic absorbers have become a solution for absorbing unwanted electromagnetic waves. This research proposed a lightweight and flexible electromagnetic absorber produced from natural rubber filled with conductive carbon black (CCB) and Fe_3_O_4_. The effect of CCB, Fe_3_O_4_, and a combination of CCB and Fe_3_O_4_ as a hybrid filler on foam morpholog, electromagnetic reflectivity, tensile strength, and compression set properties were investigated. In addition, the effect of the alternating layered structure of CCB and Fe_3_O_4_ on electromagnetic absorption was investigated. The results indicated that the composite foam exhibited an interconnected network structure that enhanced the electromagnetic attenuation in the absorber. CCB increased the electromagnetic absorption of the foam, whereas Fe_3_O_4_ had less of an effect. The foam filled with the hybrid filler at the CCB/Fe_3_O_4_ ratio of 8/2 exhibited excellent electromagnetic absorption. The composite foam had a higher tensile modulus and higher strength compared to neat foam. The addition of CCB decreased the compression set; however, the compression set was improved by the incorporation of Fe_3_O_4_. Composite foams filled with hybrid filler can serve as highly efficient electromagnetic absorbing materials.

## 1. Introduction

Electromagnetic pollution has become a severe concern owing to rapid advancements in telecommunication and wireless electronic devices. Electromagnetic pollution is caused by electromagnetic radiation emitting from electronic devices such as smartphones as well as from wireless and communication devices. The electromagnetic radiation from these devices can interfere with other electronic devices, leading to disturbance, interference, malfunction, and the leakage of information [1]. Furthermore, electromagnetic radiation can endanger the health of human beings, especially when subjected to it for a long period of time [2,3]. To solve these problems, electromagnetic shielding materials are used to absorb unwanted electromagnetic waves. The primary mechanism of electromagnetic shielding is reflection [4,5]. The principle is based on the simple reflection of electromagnetic radiation from the surface of the shielding material. A material that can reflect electromagnetic radiation must be able to conduct an electric current. Metal is the most common material used for electromagnetic reflection applications due to the high amount of free electrons. However, the usage of traditional metals as electromagnetic shielding materials is restricted by their high density, low flexibility, and corrosiveness. Several attempts have been carried out to develop polymer materials due to their lightweight, flexibility, cost-effectiveness, and chemical and corrosion resistance [6,7,8]. However, polymers are electrically insulating and transparent to electromagnetic radiation. The efficiency of the electromagnetic absorption of a polymer can be modified by dispersing different types of fillers with high dielectric permittivity and/or magnetic permeability into the polymer matrices [9,10,11]. In recent years, magnetite (Fe_3_O_4_) has attracted attention in electromagnetic shielding applications owing to its high value of permeability, which allows electromagnetic waves to enter and dissipate through magnetic loss [12,13,14]. Moreover, the ultrasmall size of Fe_3_O_4_ can greatly increase interfacial polarization because multiple reflections of electromagnetic waves can be achieved at the surface [15]. The combination of Fe_3_O_4_ with other components has been demonstrated to boost electromagnetic absorption. For instance, Liu et al. prepared TiO_2_/Ti_3_C_2_T_x_/Fe_3_O_4_ composites using a hydrothermal reaction. By tuning the ratio of [TiO_2_/Ti_3_C_2_T_x_]/[Fe_3_O_4_], the composites demonstrated extreme microwave absorption [15]. Nevertheless, the absorption bandwidth was narrow, and this problem should be addressed before use in practical applications. Conductive carbon black (CCB) is widely used for electromagnetic absorption applications because CCB has very high electrical conductivity, which enhances the dissipation of electromagnetic waves. In addition, its high specific surface area benefits the filler–matric interaction. Work by Salayong et al. [16] introduced an electromagnetic absorber made from natural rubber filled with CCB. Their work indicated that the performance of electromagnetic absorption increased as the amount of CCB increased. The natural rubber filled with 50 wt% CCB exhibited low electromagnetic reflectivity, which was close to that of a commercial absorber. Pongmuksuwan et al. [17] investigated the influence of three different types of carbon black, namely CCB and conventional carbon black types N330 and N660, on the electromagnetic reflectivity of natural rubber foams. The results revealed that the electromagnetic reflectivity of the absorber comprising CCB was significantly lower than those with N330 and N660. Al-Hartomy et al. [18] compared the dielectric properties of natural rubber filled with furnace carbon black type N220 and CCB. The results revealed that at equal concentrations, the rubber compound filled with CCB had a dielectric permittivity value that was higher than those filled with N220. According to the literature, effective electromagnetic absorbing materials should satisfy two criteria: (1) excellent impedance matching between the electromagnetic absorber and the environment and (2) strong incident electromagnetic wave attenuation in the absorber. In the former criteria, the concept of impedance matching is determined by the relationship between complex permittivity and complex permeability. For instance, if absorbing materials only have complex permittivity, then most of the incident waves will be reflected off according to the high surface resistance. On the contrary, if the absorbing materials only have complex permeability, then the incident waves pass through the absorber without any dissipation [19]. Therefore, the combination of carbon-based fillers with magnetic fillers offers a great opportunity to tune permeability and permittivity to obtain effective impedance matching. Kumari et al. [20] reported improvements in impedance matching using the conductive material Poly(3,4-ethylenedioxythiophene) (PEDOT) on the surface of an rGO/NiCoFe_2_O_4_ composite. The resultant composites exhibited the synergy of dielectric and magnetic loss on the attenuated behavior of the microwaves in the absorber. Dalal et al. [21] fabricated Ni_0.5_Co_0.5_Fe_2_O_4_/RGO nanocomposites for use as microwave absorbers. The permeability and permittivity of the absorber could be controlled by controlling the content of RGO in the composite. Liu et al. reported on a superior microwave absorber prepared using a combination of magnetic nanoparticles (Co doping in Ni–Zn ferrite (CNZF)) and dielectric nanosheets (graphene (GN)). Besides impedance matching, the lags of the induced charges originating from the ferrite–ferrite, ferrite–GN, and GN–GN interfaces led to the relaxation and transformation of electromagnetic energy to thermal energy [14]. In the latter criteria, multiple reflections are an important mechanism of electromagnetic wave attenuation in the absorber. The multiple reflections refer to the electromagnetic radiation reflected from various surfaces and the inhomogeneities in the absorbing material. Materials with a high specific surface area, e.g., foam or porous materials, exhibit good multiple reflection ability [22,23]. The cellular structure provides numerous inner transmissions and multiple internal reflections; as a result, the amplitude of the incident electromagnetic wave is attenuated. Natural rubber latex (NRL) is among the best candidate materials to promote a foam structure. NRL can be whipped into a froth with the assistance of foaming agents. In addition, NRL can be compounded with vulcanizing agents, resulting in better flexibility and elasticity. Moreover, NRL is also considered to be an economic plant in Thailand [24].

This work aims to develop a high-performance electromagnetic absorber consisting of NRL as the matrix and CCB and Fe_3_O_4_ as the fillers. The incorporation of CCB and Fe_3_O_4_ in NRL as a hybrid filler offers a great opportunity to improve the electromagnetic absorption of NRL. The NRL foam structure was introduced for the multiple reflections of the electromagnetic waves in the absorber. The effect of the CCB and Fe_3_O_4_ contents and the ratio of CCB/Fe_3_O_4_ as a hybrid filler on the foam morphology and electromagnetic absorption were investigated. In addition, a multilayer structure consisting of alternating layers of CCB and Fe_3_O_4_ was fabricated. The electromagnetic absorption of the multilayer structure and the single-layer structure with the hybrid filler was compared. Furthermore, the tensile properties and compression sets of the composite foams were investigated.

## 2. Materials and Methods

### 2.1. Materials

High-ammonia NRL with a dried rubber content of 60% and all of the other chemical reagents: sulphur, zinc diethyldithiocarbamate (ZDEC), zinc mercaptobenzothiazole (ZMBT), Wingstay L antioxidant, zinc oxide (ZnO), Diphenylguanidine (DPG), sodium silicofluoride (SSF), and potassium oleate (K-oleate), were purchased from the Rubber Research Institute of Thailand (Bangkok, Thailand). The CCB (ENSACO250G) was purchased from Global connections PCL. (Bangkok, Thailand). The CCB had a particle size of 35 nm, a specific surface area (BET) of 65 m^2^/g, and an oil absorption number of 190 mL/100 g. Fe_3_O_4_ powder was purchased from Macking Green Herbal Store (Shenzhen, China).

### 2.2. Preparation of Single Layer NRL Composite Foams

**Figure 1:**
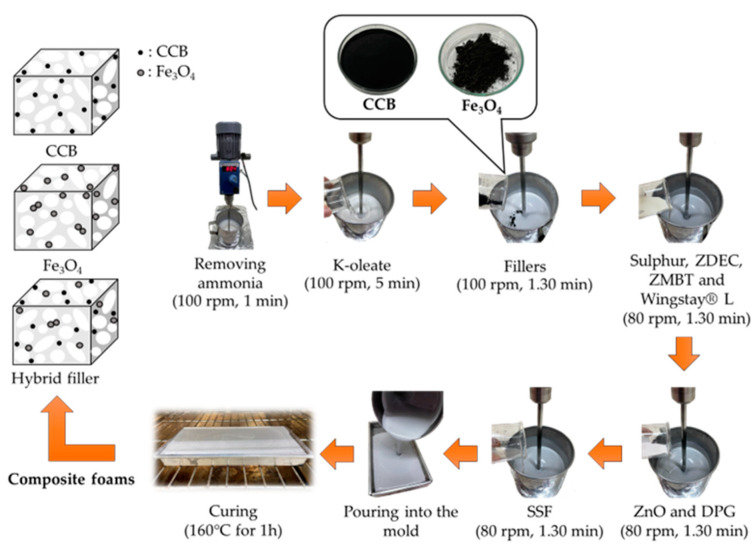
The preparation of composite foam.

The composite foam was prepared as illustrated in Figure 1. Solid reagents such as sulphur, ZDEC, ZMBT, ZnO, CCB, and Fe_3_O_4_ were prepared in dispersion form using ball-milling at a speed of 65 rpm for 24 h before mixing with NRL. The quantities of the reagents used in the NRL foam composites are listed in Table 1. The NRL was stirred using a high-speed stirrer with a rotation speed of 100 rpm for 1 min to remove ammonia. K-oleate (foaming agent) was added to the NRL, and the mixture was stirred continuously until the volume of the mixture became frothy. Subsequently, the CCB and Fe_3_O_4_ dispersion were added, and the mixture was stirred for 1.30 min. The CCB and Fe_3_O_4_ contents were 4, 6, 8, 10, and 12 phr. After that, the stirring speed was reduced to 80 rpm; then, a group of chemicals (sulphur, ZDEC, ZMBT, and Wingstay L) was added, and the mixture was stirred for 1.30 min. After that, ZnO and DPG were added as the secondary gelling agents, and stirring continued for 1.30 min. Subsequently, SSF was added as the primary gelling agent, and the stirring was maintained until the mixture was homogeneous. The mixture was then poured into aluminum molds (110 × 110 × 25 mm^3^), and the lids were closed afterwards. Finally, the samples were heated at 160 °C in a hot air oven for 1 h. According to our previous work, the optimum thickness for NRL filled with CCB to achieve electromagnetic absorption is 25 mm [25]. Therefore, in this study, the thicknesses of the absorbers were fixed at 25 mm.

**Figure 2:**
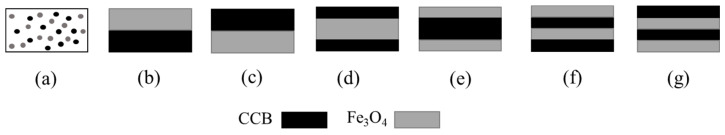
The layered-structure foams consisting of CCB and Fe_3_O_4_ layers: (**a**) a single layer with CCB and Fe_3_O_4_ fillers; (**b**,**c**) two layers; (**d**,**e**) three layers; and (**f**,**g**) four layers.

In this study, the electromagnetic absorption levels of single and multilayer foams are compared. In the case of two layers, after the mixtures of the NRL filled with CCB and Fe_3_O_4_ were prepared, the initial layer was poured into a mold, and it was allowed to set for 1.30 min. After that, the second layer was poured into a mold, and the mixture was then heated in an oven at 160 °C for 1 h. In the case of three and four layers, the bottom layer, interlayer, and top layer were poured into a mold layer by layer. After finishing each layer, the mixture was allowed to set for 1.30 min before the next layer was poured. Then, the mixture was heated in an oven at 160 °C for 1 h to obtain the multilayer composite foams. The single-layer and layered-structure foams made from a combination of CCB and Fe_3_O_4_ with two, three, and four layers are depicted in Figure 2.

### 2.3. Characterization

#### 2.3.1. Cell morphology and Cell Size

The morphologies of the composite foams were determined by scanning electron microscopy (SEM, JEOL JSM-6610LV, Oxford X-Max 50, Tokyo, Japan) at 15 kV. The fracture surfaces of the composite foams were obtained after a 2 min immersion in liquid nitrogen. The cell sizes of the composite foams were quantified using ImageJ version 1.5i open-source image processing software (the Laboratory for Optical and Computational Instrumentation, University of Wisconsin, Madison, WI, USA). The average cell size was quantified based on the average cell size of 100 cells.

#### 2.3.2. Electromagnetic Reflectivity of Composite Foam

**Figure 3:**
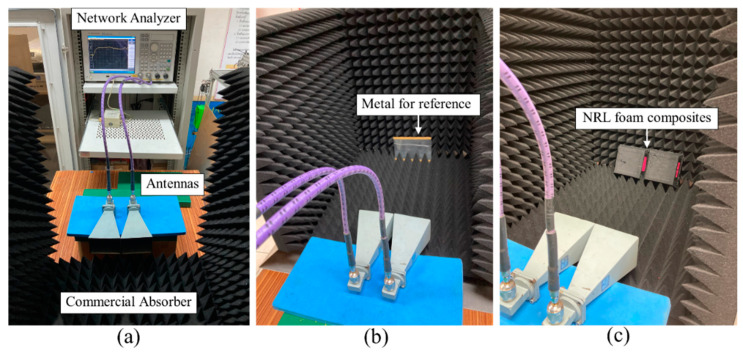
Measurement setup of reflectivity in the frequency range of 8–14 GHz. (**a**) Setup of the network analyzer used for measurement; (**b**) depiction of the metal only for reference; (**c**) proposed EM absorber with metal.

Figure 3 shows an experimental setup for the free space measurement methods used for the reflectivity measurements of the electromagnetic wave absorbers. The electromagnetic reflectivity performance of the composite foams was measured by a vector network analyzer (Agilent E5071C, Keysight, Colorado Springs, CO, USA) coupled with two horn antennas. A metal plate was used as a reference because all electromagnetic waves can be reflected off of metal [11]. The metal plate was located in the test chamber; then, the sample was placed in front of the metal plate, and the distance between the horn antennas and the absorber was set at 50 cm. Commercial pyramidal absorbers were employed around the sample to eliminate electromagnetic interference from the surrounding environment. The samples were evaluated by directly measuring the reflection loss (RL) in the frequency range of 8–14 GHz. Electromagnetic wave absorption was performed on five samples per formula.

#### 2.3.3. Tensile Test

The tensile properties of the foam composite absorbers were determined using a universal testing machine (Instron model 1123, Instron, Canton, MA, USA) according to ASTM D412 [26,27]. The specimen was cut into a dumbbell shape (115 × 33 × 2 mm^3^). The tensile tests were conducted at 25 °C, with a crosshead speed of 500 mm/min and a gauge length of 33 mm. The tensile strength, tensile modulus, and elongation at break were averaged from five specimens.

#### 2.3.4. Compression Set

The compression set is the ability of a rubber specimen to retain its elastic properties after being subjected to prolonged compressive force at constant strain. The compression sets of the NRL foam composites were determined under the conditions outlined in ASTM D1055-97. The samples were cut into a cubic shape (30 × 30 × 20 mm^3^). The samples were squeezed between the two metal plates of the compression apparatus and were compressed to 50 ± 1% of their original thickness. Within 15 min, the compressed specimens and the compression apparatus were placed in an air oven for a period of 22 h at a test temperature of 70 ± 2 °C. Then, the specimens were removed from the compression apparatus and were allowed to recover for 30 min; then, the thickness of the specimens was measured. The compression set was calculated according to Equation (1) [28,29]:

(1) $$\mathrm{Compression}\;\mathrm{set}\;\left(\%\right)=\left[\frac{T_{0}-T_{i}}{T_{0}-T_{n}}\right]\times 100$$

where $T_{0}$ is the original thickness (mm);

$T_{i}\;$ is the 10 mm spacer bar used to maintain the compression deflection;
$T_{n}$ is the final thickness of the foam composite.

## 3. Results and Discussion

### 3.1. Morphology of CCB and Fe_3_O_4_ Particles

**Figure 4:**
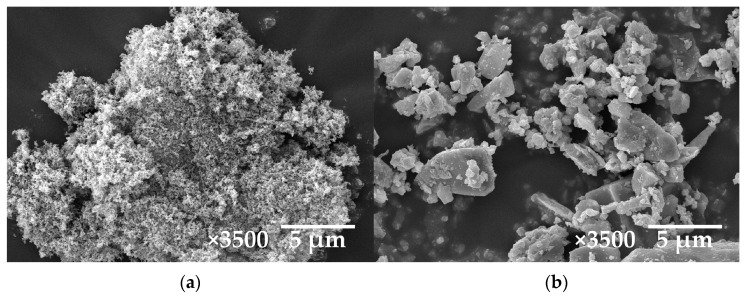
The SEM micrographs of (**a**) CCB and (**b**) Fe_3_O_4_.

Figure 4 shows the SEM micrographs of CCB and Fe_3_O_4_. As shown in Figure 4a, CCB has fine particles, with the complexity of the arrangements in the agglomerates resulting in a relatively high porosity. The high-porosity structure makes it possible to establish a carbon network, resulting in the increment of the transmission channel of the electromagnetic waves. At the same time, the high-porosity structure increases the specific surface area, which could be helpful to enhance filler–rubber interaction. As shown in Figure 4b, the Fe_3_O_4_ particles are of an irregular and stone-like shape, with a particle size range of 1–30 µm.

### 3.2. Cell Characteristics

**Figure 5:**
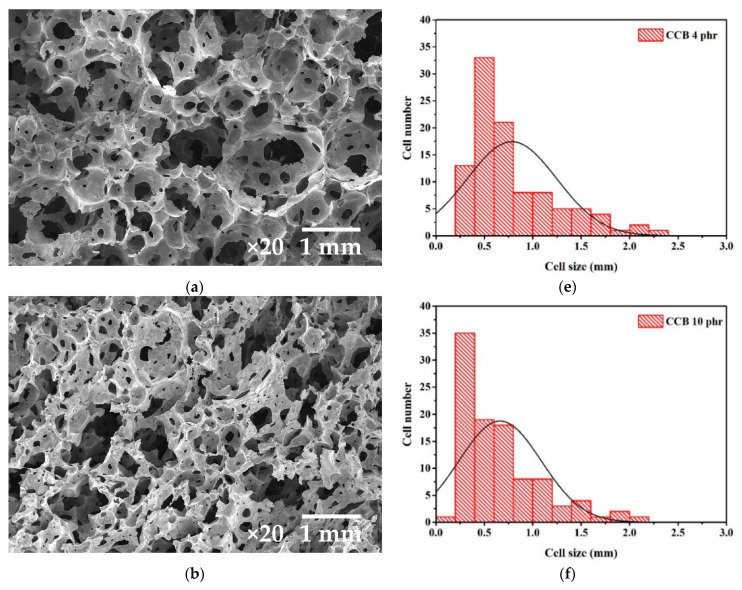

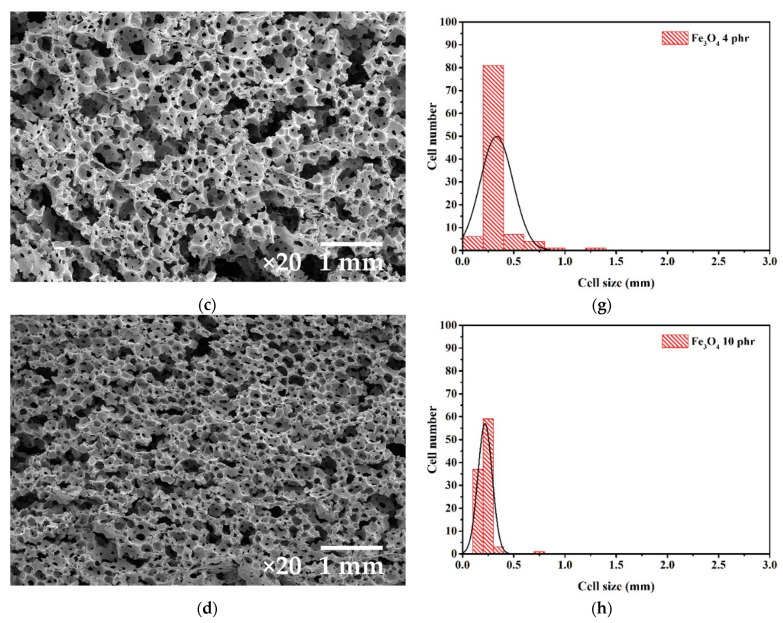
Morphology of the NRL foam composites with different contents of CCB and Fe_3_O_4_; (**a**–**d**) cellular morphology and (**e**–**h**) cell size distribution.

Figure 5 presents the SEM micrographs and cell size distributions of composite foams with different CCB and Fe_3_O_4_ contents. The cell structures of the composite foams were found to form opened cell structures that exhibit an interconnected network of cells. The formation of open cell structures can reduce the density of the composite foam and is an effective structure for electromagnetic absorption. The introduction of open cells provides numerous transmission channels for the incident electromagnetic waves. The high specific area and void give rise to scattering, multiple reflections, and the absorption of electromagnetic waves, which results in the promotion of the electromagnetic attenuation in the absorber. As seen in Figure 5, the cells of the foam containing 4 phr CCB are spherical and have smooth interfacing surfaces with the cell wall. The cell size ranged from 250 to 2500 µm, with a mean cell size of 780 µm. The cell size in composite foams tends to shift toward a smaller cell size with an increase in the CCB content to 10 phr. Moreover, the cell walls decrease, resulting in increasing cell windows, which increase cell connectivity. The decrease in cell size at high CCB contents is due to the strong interface force and entanglement between CCB and the natural rubber matrix acting as the physical crosslinking points, which hinders the molecular chains’ expansion during foam growth. As the CCB content increases, the number of interactions increases, which obstructs the bubble expansion, resulting in the formation of smaller bubbles. Moreover, the higher amount of CCB increases the rubber viscosity, which limits bubble growth. These results agree with classical nucleation theory, which states that the increment in the number of smaller cells can be ascribed to the increase in polymer viscosity [30,31,32]. In the case of NRL filled with Fe_3_O_4_, the foams exhibit fine and uniform cell sizes compared to those containing CCB. The cell size and cell distribution of the foams containing Fe_3_O_4_ follow a similar trend as seen with those containing CCB, with the cell size and cell distribution decreasing as the Fe_3_O_4_ content increases. However, the foam containing Fe_3_O_4_ shows a narrower cell size distribution as well as a higher fraction of smaller cells compared to those containing CCB.

**Figure 6:**
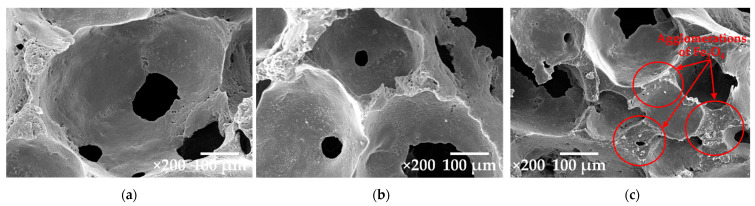
SEM images of the composite foams containing hybrid fillers with different contents of CCB/Fe_3_O_4_: (**a**) 9/1; (**b**) 8/2; and (**c**) 7/3.

Figure 6 shows the SEM micrographs of the composite foams containing hybrid fillers of CCB and Fe_3_O_4_. The SEM observations demonstrate the open cell structures and different contents of CCB and Fe_3_O_4_, exhibiting different foam morphologies. As the content of CCB decreases and the content of Fe_3_O_4_ increases, the cell sizes decrease, whereas the thicknesses of the cell wall increase. Moreover, the agglomeration of Fe_3_O_4_ particles can be observed for the foam containing CCB/Fe_3_O_4_ at a ratio of 7/3. The morphological properties of foams and agglomerations with Fe_3_O_4_ play important roles in electromagnetic absorption and mechanical properties (details are presented in the Section 3.3).

### 3.3. Electromagnetic Reflectivity

**Figure 7:**
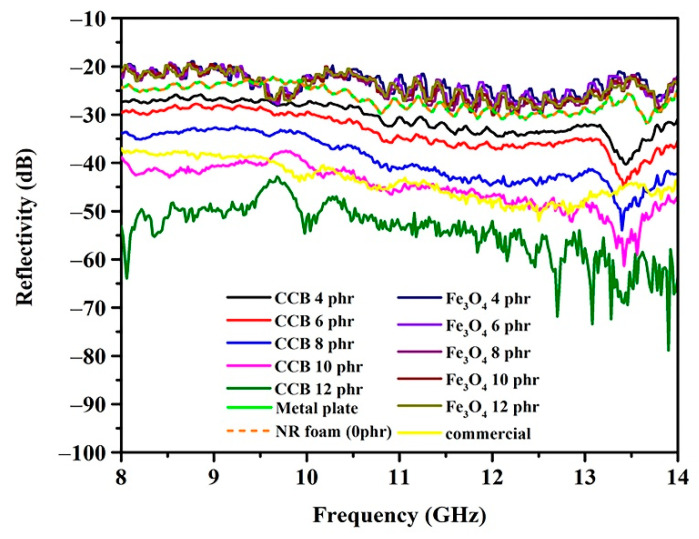
Reflection loss curves of composite foams with different contents of CCB and Fe_3_O_4_ in a frequency range from 8 to 14 GHz.

Figure 7 shows the reflectivity differences between the transmitting and receiving antennas of composite foams containing different contents of CCB and Fe_3_O_4_. The reflectivities of the commercial absorber and metal plate were plotted for comparison purposes. Generally, natural rubber is a typical electric insulator, as seen in Figure 7, where the reflectivity of neat foam is close to that of the metal plate, indicating that natural rubber is not able to absorb electromagnetic waves. Therefore, electromagnetic waves can penetrate the metal plate and then be reflected onto the receiving antenna. The foam containing CCB shows a decrease in the amplitude of the reflectivity at all of the frequency ranges of the incident waves. This is due to the excellent electrical conductivity of CCB, which is an important criterion for electromagnetic absorption. The reflectivity of the electromagnetic waves progressively decreases as the CCB content increases. At low a CCB content, a small number of conductive particles are in contact with each other; thus, the electrical conductivity increases slightly, and consequently, the reflectivity decreases slightly. At a high CCB content, the conductive particles are close to each other, and electrons can easily transfer between particles, increasing the number of conductive pathways. The conductive pathways act to dissipate the mobile charge carriers, leading to higher electromagnetic dissipation during absorption. Moreover, as seen in Figure 5, increasing the CCB content results in an increase in cell connectivity, which enhances the conductive pathways in the absorber. As a result, electrical conductivity and electromagnetic absorption increase. It can be seen in Figure 6 that foam containing a CCB content of 10 phr exhibits reflectivity close to that of the conventional absorber, whereas foam containing a CCB content of 12 phr exhibits lower reflectivity compared to that of the commercial absorber. This result indicates the feasibility of using natural latex foam filled with CCB as an electromagnetic absorber. In the case of the foam containing Fe_3_O_4_, the reflectivity of the foam for any given Fe_3_O_4_ content level is close to that of neat NRL, and the reflectivity remains unchanged, even when the content of Fe_3_O_4_ is increased up to 12 phr. It is known that Fe_3_O_4_ is a magnetic material with high magnetic permeability. However, poor electromagnetic impedance matching and a small dielectric constant of Fe_3_O_4_ limit the propagation of the electromagnetic waves [33,34,35].

### 3.4. Influence of Hybrid Filler on the Electromagnetic Absorption

**Figure 8:**
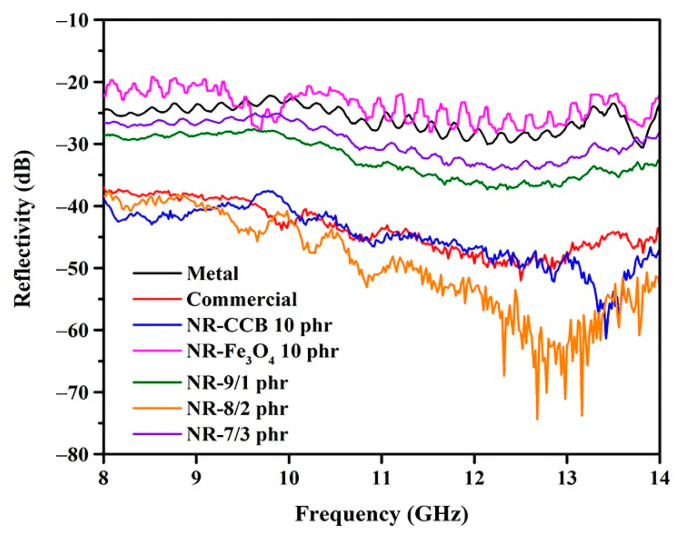
Reflectivity of composite foams with different contents of CCB and Fe_3_O_4_.

The goal of combining Fe_3_O_4_ and CCB as a hybrid filler is to obtain dielectric and magnetic properties for the absorber, which contribute to the improvement of electromagnetic absorbing properties. Figure 8 shows the reflectivity of the composite foams containing hybrid fillers with different contents of CCB/Fe_3_O_4_. It is noted that the amount of total filler in all of the samples was kept constant at 10 phr. The foam containing a CCB content of 12 phr exhibits the lowest electromagnetic reflectivity. However, this is according to the cost-effective and lightweight applications of the absorbers. Therefore, this study selected the CCB content of 10 phr, which exhibits reflectivity close to that of conventional absorbers, to further study the effects of hybrid filler on electromagnetic reflectivity. The foam containing a CCB content of 9 phr and an Fe_3_O_4_ content of 1 phr shows higher reflectivity than the foam containing CCB 10 phr and reflectivity that is close to that of the foam containing CCB at 8 phr. This result indicates that the addition of1 phr Fe_3_O_4_does not result in a significant improvement in electromagnetic absorption. When the content of CCB is 8 phr and the content of Fe_3_O_4_ is 2 phr, the reflectivity of the composite foam was reduced. This was described as being due to the increase in Fe_3_O_4_ resulting in increased magnetic loss ability values. As mentioned in the literature [36,37], the concept of impedance matching is determined by the relationship between dielectric and magnetic loss ability. CCB exhibits some dielectric loss ability; however, their imaginary complex permeability is too low. The addition of Fe_3_O_4_ increases the magnetic loss ability of the absorber; therefore, the characteristic impedance is improved effectively. Furthermore, the decrease in reflectivity was described as being due to the high interface area between the CCB and Fe_3_O_4_ particles, which increases as the Fe_3_O_4_ content increases. The increase in the interface area between the CCB and Fe_3_O_4_ particles results in an increase in interfacial polarization, which occurs on the CCB particles with relatively high conductivity. This leads to the accumulation of charges at the interfaces and the generation of dipoles on the Fe_3_O_4_ particles. Thus, interfacial polarization and the associated space charge relaxation processes contribute to the electromagnetic shielding performance. In hybrid composites, Fe_3_O_4_ acts as a polarized center in the presence of electromagnetic radiation, which provides space for better absorption. Moreover, it is easy to observe the presence of multiple resonance peaks, which are the result of resonance at the interface introduced by the heterostructure along with polarization loss [38]. However, the reflectivity of composite foam increases when the content of CCB is 7 phr and the content of Fe_3_O_4_ is 3 phr. The higher the electromagnetic reflectivity is probably due to the agglomeration of Fe_3_O_4_, as seen in Figure 6. Moreover, when the content of CCB decreases, the perfect conductive networks of CCB become inferior, resulting in the decrement of the transmission channel of the electromagnetic waves.

### 3.5. Influence of Multilayers Structure on the Electromagnetic Absorption

**Figure 9:**
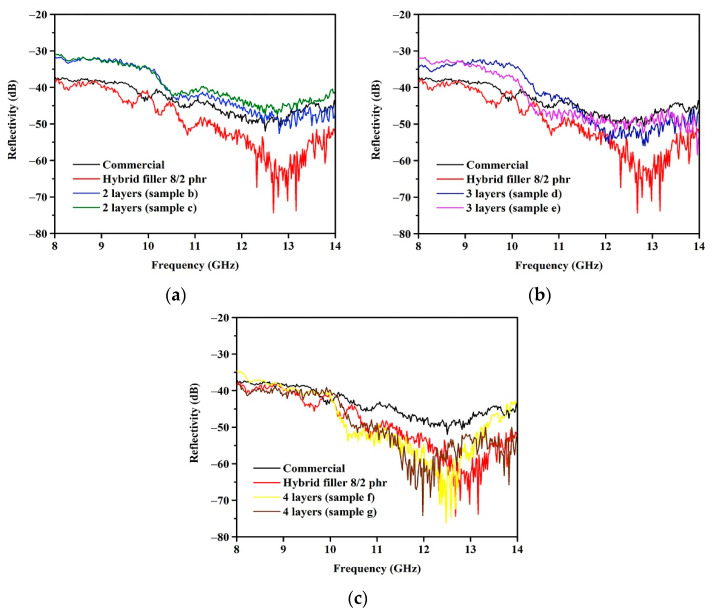
Reflectivity of composite foams with different layers of CCB and Fe_3_O_4_: (**a**) 2 layers; (**b**) 3 layers; and (**c**) 4 layers.

The goal of fabricating a multilayer absorber was to find a set of layers that minimizes electromagnetic reflection and to compare the performance of multilayer and single-layer structures during electromagnetic absorption. The reflectivity of the layered-structure foams with two, three, or four layers made with a combination of CCB and Fe_3_O_4_ are depicted in Figure 9. The reflectivities of the single-layered foams containing hybrid fillers made of CCB at 8 phr and Fe_3_O_4_ at 2 phr are plotted for comparison. As seen in Figure 9, the reflectivities of the layer-structured foams are similar for the different sides, indicating that applying the incident wave from the different sides of the sample has an insignificant effect on electromagnetic absorption. The two- and three-layer foams show poor electromagnetic absorption in comparison to the single-layer foam, whereas the four-layer foam has electromagnetic absorption close to that of the single-layer foam containing the hybrid fillers composed of CCB and Fe_3_O_4_. In the case of a single-layer structure, the electromagnetic waves are attenuated by the high electrical conductivity of CCB and the magnetic permeability of the incorporated Fe_3_O_4_, resulting in interfacial polarization between the CCB and Fe_3_O_4_ particles. However, in the case of multilayered foam, the effect of interfacial polarization is minimized. The interfacial polarization in multilayer foam can only occur at the interface between the CCB and Fe_3_O_4_ layers. The higher the number of interface layers, the more that interfacial polarization can occur. Therefore, the reflectivity of four-layer foam is close to that of a single-layer foam containing hybrid fillers.

### 3.6. Tensile Test

**Figure 10:**
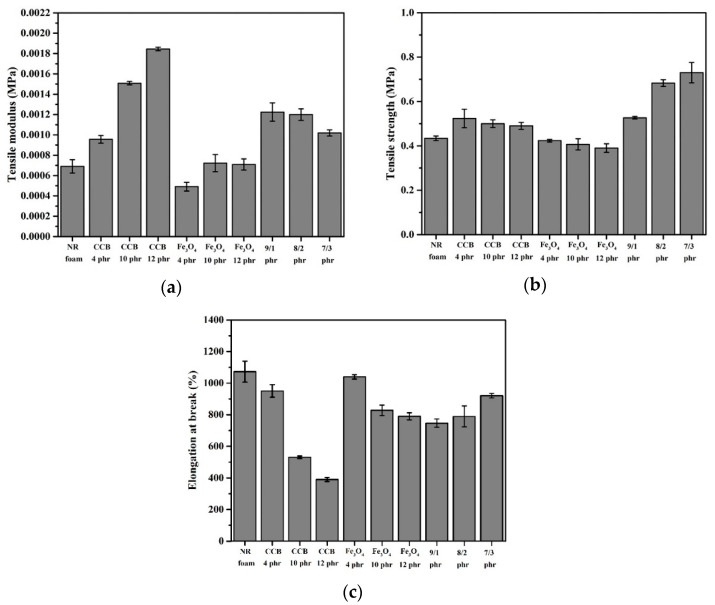
Tensile properties of NR foam composites with different contents of CCB and Fe_3_O_4_ and different CCB/Fe_3_O_4_ ratios: (**a**) tensile modulus, (**b**) tensile strength, and (**c**) elongation at break.

Figure 10 shows the tensile properties in terms of the tensile moduli, tensile strengths, and elongations at the break of the composite foams containing CCB, Fe_3_O_4_, and hybrid fillers comprising CCB:Fe_3_O_4_ at various weight fraction ratios. The presence of CCB significantly improves the tensile modulus and tensile strength of composite foams compared to neat foam. The tensile modulus of composite foams continuously increases as the CCB content increase, whereas tensile strength is independent of the CCB content. Carbon black is known as an effective reinforcing filler. The introduction of carbon black into rubber results in a restriction in chain mobility, resulting in an increment in the tensile modulus and tensile strength [27,39]. However, the tensile strength of the composite foams containing CCB contents higher than 4 phr remains unchanged. This is due to the dilution effect, which is caused by an insufficient amount of rubber to accommodate the filler at high CCB loadings. In the case of composite foams containing Fe_3_O_4_, the composite foams containing an Fe_3_O_4_ content of 4 phr exhibit a lower tensile modulus compared to neat foam. This is associated with the lack of interfacial adhesion between Fe_3_O_4_ and the rubber matrix. Fe_3_O_4_ has no chemical functional groups that can react with rubber macromolecules. However, the tensile modulus of composite foam increases when the content of Fe_3_O_4_ increases to 10 phr and levels off at when the Fe_3_O_4_ content is 12 phr. It is known that the tensile modulus is an indicator related to the stiffness of materials. The increase in the Fe_3_O_4_ content leads to an increase in the stiffness of composite foam. However, the unchanged tensile modulus of the composite foams containing Fe_3_O_4_ at 12 phr is probably due to the competitive effects occurring simultaneously; the stiffening effect of Fe_3_O_4_ increases the modulus of composite foam, whereas poor adhesion between Fe_3_O_4_ and the rubber matrix impacts chain entanglement, resulting in a decrease in the modulus of composite foam.

Considering the foam morphology in Figure 6, it can be seen that the foam containing CCB exhibits larger cell sizes compared to those containing Fe_3_O_4_. The large cell sizes exhibit poor tensile properties due to the stress concentration induced by their large size. Furthermore, the large cells act as cracks or defects during testing, resulting in tensile strength reductions [40]. However, in this study, the foams containing CCB exhibit a higher tensile modulus and tensile strength compared to the foam containing Fe_3_O_4_. This is due to the better compatibility and the lower particle size of CCB, which provide better interaction between the rubber and the interface of the filler, leading to a higher reinforcing effect. Figure 10c shows the elongations at the break of the composite foams, which were found to be decreased as the filler content increased. The addition of CCB has a more pronounced effect on the decrease in the elongation at break when the CCB content is higher than 4 phr. The elongation at break decreases sharply for the composite foam containing CCB at 10 phr. The decrease in the elongation at break is because the addition of filler restricts the mobility of rubber molecules. This effect becomes stronger when there is an increase in CCB loading. The foam containing CCB exhibits lower elongation at break when compared with those containing Fe_3_O_4_ at a similar filler content. For instance, the elongation at break of the foam containing CCB at 10 phr is about 550%, whereas that of the foam containing Fe_3_O_4_ at 10 phr is about 800%. This is because the high interfacial interaction between the CCB particles and the rubber matrix makes composite foam harder and more rigid. Moreover, as seen in Figure 2, CCB has smaller particle sizes, leading to a higher specific surface area and stronger restriction of the rubber chain mobility. As a result, the composite foams containing CCB tend to break at a lower elongation.

In the case of foams containing hybrid fillers, the tensile moduli of the composite foams containing hybrid fillers with CCB/Fe_3_O_4_ ratios of 9/1 and 8/2 are not different. The tensile modulus starts to decrease for those foams containing CCB/Fe_3_O_4_ at a ratio of 7/3. The decrease in modulus is caused by the decrease in the reinforcing effect originating from the reduction in the CCB content. The decrease in the reinforcing effect is less pronounced in the case of the tensile strength and elongation at break. The tensile strengths and elongations at break continuously increase as the ratio of CCB/Fe_3_O_4_ decreases. It is known that the morphology of foam plays an important role in influencing mechanical properties. As seen in Figure 6, when the content of CCB decreases and the content of Fe_3_O_4_ increases, the foams exhibit a higher number of cells with thicker cell walls. The increase in cell wall thickness increases the load-bearing area in the sample, resulting in an increase in tensile strength and elongation at break.

### 3.7. Compression Set

**Figure 11:**
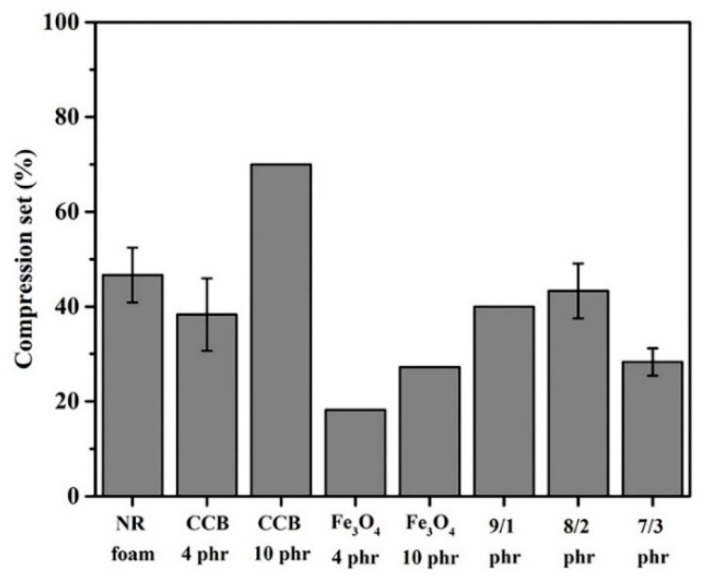
Compression set of composite foams with different contents of CCB and Fe_3_O_4_ and different CCB/Fe_3_O_4_ ratios.

Figure 11 shows the compression set results of the composite foams containing CCB, Fe_3_O_4_, and hybrid fillers of CCB and Fe_3_O_4_ at various weight fraction ratios. The compression set corresponds to the ability of a material to recover to its original thickness after deformation under a compressive force. A low compression set value indicates the good recoverability of composite foam after load removal. It can be observed that the compression set of the foam containing CCB at 4 phr did not change significantly compared to that of neat foam. This is because the low content of CCB has little effect on the mobility of the rubber chains. The compression set increases as the content of CCB increases to 10 phr. The compression set of foam containing CCB at 10 phr was 25% higher than that at 4 phr, indicating that the ability of the composite to return to its original shape had reduced as the content of the CCB increased. It is known that the high interfacial interaction between the CCB particles and natural rubber causes an increase in the chain entanglement and crosslink density, which decreases the mobility of rubber chains [41,42]. During compression, the entangled chains and crosslinks are mainly involved in resisting the compression force. Consequently, this resistance can cause damage to some crosslinks. After removing the compression force, the number of crosslinks responsible for strain recovery is less than the number of crosslinks required to resist compression. As a result, the specimen did not recover to its original thickness. On the contrary, the foams containing Fe_3_O_4_ showed a lower compression set compared to neat foam and those foams containing CCB. The addition of Fe_3_O_4_ should have less of an impact on the compression set because Fe_3_O_4_ is an inorganic filler that has limited interaction with the rubber matrix. However, in this study, the addition of Fe_3_O_4_ decreases the compression set of composite foam. This is because the compression set is influenced by both filler loading and foam morphology. As presented in Figure 6, the addition of Fe_3_O_4_ results in a decrease in the average cell size as the cell density increases. The denser the materials, the lower the compression set that is obtained will be. The compression set of foam containing Fe_3_O_4_ increases with as the Fe_3_O_4_ content to 10 phr. This is because the high rigidity of the Fe_3_O_4_ particles increases the stiffness of the composite foams, resulting in the reduction of elasticity. Since the compression set is a property used to indicate the degree of elasticity, the increase in the compression set values implies that the materials have less elasticity.

In the case of composite foams containing a hybrid filler, the compression set is unchanged at CCB/Fe_3_O_4_ content ratios of 9/1 and 8/2 phr. The compression set starts to decrease at the CCB/Fe_3_O_4_ content ratio of 7/3 phr. The decrease in the compression set can be explained by two reasons. Firstly, the increase in the stiffness of the specimens is due to the high rigidity of Fe_3_O_4_. Secondly, the decrease in the CCB content can be attributed to a decrease in rubber–filler interaction. As a result, the mobility of rubber molecules increases. Comparing the compression set of the foams containing CCB and Fe_3_O_4_, the foams containing hybrid filler for any given content level show lower compression set values than those containing CCB but that are higher than those containing Fe_3_O_4_. This result indicates that the addition of Fe_3_O_4_ can improve the compression set of those foams containing CCB.

## 4. Conclusions

NRL composite foams filled with CCB and Fe_3_O_4_ were developed for use as electromagnetic absorbers. The effects of CCB, Fe_3_O_4_, and the combination of CCB and Fe_3_O_4_ as a hybrid filler on the electromagnetic reflectivity and mechanical properties of composite foams were investigated. The results demonstrated that the composite foams exhibit an interconnected network, which is an effective structure for electromagnetic absorption. The reflectivity of electromagnetic waves decreased as the CCB content increased, whereas the reflectivities of the foams filled with Fe_3_O_4_ remained unchanged. In the case of the hybrid CCB/Fe_3_O_4_ composite, excellent electromagnetic reflectivity was obtained by the synergistic effect of CCB and Fe_3_O_4_ at the CCB/Fe_3_O_4_ ratio of 8/2. The composite foams filled with hybrid filler can be used as efficient electromagnetic shields, with absorption dominated in the frequency range of 8–14 GHz. The single-layer foams filled with hybrid filler showed efficient electromagnetic absorption compared to multilayer foam. The addition of CCB increased the tensile modulus and tensile strength of NRL foam, whereas the addition of Fe_3_O_4_ had less of an effect on the tensile properties. The foam with a hybrid filler exhibited a higher tensile modulus and higher tensile strength compared to that of neat NRL foam; however, the increase in the Fe_3_O_4_ content in the hybrid filler deteriorates the tensile modulus and the tensile strength of composite foam. The addition of CCB increases the compression set value of NRL foam, whereas the compression set value decreased with the addition of Fe_3_O_4_. The composite foam with hybrid filler exhibits compression set values close to those of neat NRL foam. The composite foams filled with hybrid filler can serve as highly efficient electromagnetic absorbing materials with low reflection characteristics.

## Figures and Tables

**Table 1:** Compositions of the NRL foam composites.

Ingredients	Concentration (phr)
NR latex	100
K-oleate (10%)	1.5
CCB	0, 4, 10, 12
Fe_3_O_4_	0, 4, 10, 12
Sulphur (50%)	2
ZMBT (50%)	1
ZDEC (50%)	1
Wingstay L (50%)	1
ZnO (50%)	5
DPG (33%)	1
SSF (12.5%)	0.6

## Data Availability

The data presented in this study are available on request from the corresponding author.
